# 4-Acetyl-2,3,4,5-tetra­hydro-1*H*-1,4-benzodiazepine

**DOI:** 10.1107/S1600536808007927

**Published:** 2008-05-03

**Authors:** Qing-Jie Zhao, Zheng Liu, Jin Zheng, Jing-Shan Shen

**Affiliations:** a Shanghai Institute of Materia Medica, Chinese Academy of Sciences, Shanghai, 201203, People’s Republic of China

## Abstract

The title compound, C_11_H_14_N_2_O·H_2_O, crystallizes with one formula unit in the asymmetric unit. The seven-membered ring has a chair conformation with the C=O group turned away from the benzene ring. N—H⋯O and O—H⋯O hydrogen bonds are present in the crystal structure.

## Related literature

For related literature, see: Allen *et al.* (1987 ▶); Ding *et al.* (1999 ▶); Grunewald *et al.* (1996 ▶); Kim (1976 ▶).
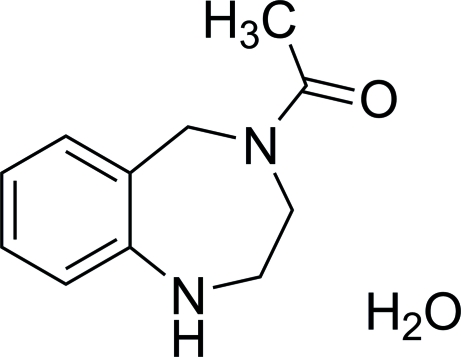


## Experimental

### 

#### Crystal data

C_11_H_14_N_2_O·H_2_O
*M* *_r_* = 208.26
Tetragonal, 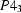

*a* = 10.8251 (8) Å
*c* = 9.4569 (14) Å
*V* = 1108.2 (2) Å^3^
*Z* = 4
Mo *K*α radiation
μ = 0.09 mm^−1^
*T* = 296 (2) K
0.20 × 0.20 × 0.15 mm

#### Data collection

Bruker APEXII CCD diffractometer
Absorption correction: none
5758 measured reflections
1043 independent reflections
972 reflections with *I* > 2σ(*I*)
*R*_int_ = 0.016

#### Refinement

*R*[*F* ^2^ > 2σ(*F* ^2^)] = 0.035
*wR*(*F* ^2^) = 0.098
*S* = 1.07
1043 reflections
140 parameters
1 restraint
H atoms treated by a mixture of independent and constrained refinement
Δρ_max_ = 0.21 e Å^−3^
Δρ_min_ = −0.20 e Å^−3^

### 

Data collection: *APEX2* (Bruker, 2000 ▶); cell refinement: *SAINT* (Bruker, 2000 ▶); data reduction: *SAINT*; program(s) used to solve structure: *SHELXS97* (Sheldrick, 2008 ▶); program(s) used to refine structure: *SHELXL97* (Sheldrick, 2008 ▶); molecular graphics: *SHELXTL* (Sheldrick, 2008 ▶); software used to prepare material for publication: *SHELXTL*.

## Supplementary Material

Crystal structure: contains datablocks I, New_Global_Publ_Block. DOI: 10.1107/S1600536808007927/om2218sup1.cif

Structure factors: contains datablocks I. DOI: 10.1107/S1600536808007927/om2218Isup2.hkl

Additional supplementary materials:  crystallographic information; 3D view; checkCIF report

## Figures and Tables

**Table 1:** Hydrogen-bond geometry (Å, °)

*D*—H⋯*A*	*D*—H	H⋯*A*	*D*⋯*A*	*D*—H⋯*A*
N1—H1⋯O1*W*^i^	0.88 (5)	2.33 (4)	3.163 (3)	158 (4)

Symmetry codes: (i) 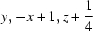; (ii) 
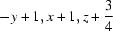
.

**Fig. 1.**
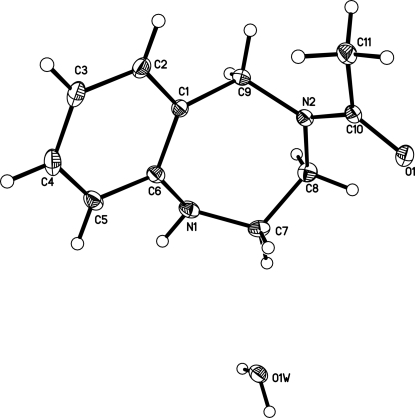
View of the molecule of (I) showing the atom-labelling scheme. Displacement ellipsoids are drawn at the 30% probability level and H atoms are shown as small spheres of arbitrary radii.

**Fig. 2.**
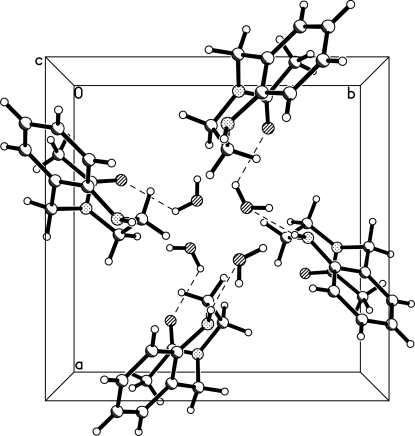
The crystal packing of (I), viewed along the c-axis. Hydrogen bonds are shown as dashed lines.

## supplementary crystallographic information

### Comment

The title molecule (Fig. 1) is an important imtermediate used to synthesize a variety of pharmaceuticals, such as inhibitors of phenylethanolamine *N*-methyltransferase (Grunewald *et al.*, 1996) and inhibitors of Farnesyltransferase (Ding *et al.*, 1999). In our recent research for exploring new methods for synthesis of benzodiazepine derivatives, 4-acetyl-2,3,4,5-tetrahydro-1*H*-1,4-benzodiazepine is synthesized in 96% yield from 2,3,4,5-tetrahydro-1*H*-1,4-benzodiazepine (Ding *et al*., 1999). We report here the crystal structure of the title compound, which crystallizes in the tetragonal space group P4(3) with one H_2_O molecule in the asymmetric unit.

A view of the molecular structure of title compound is depicted in Fig.1. The central seven-membered ring has an chair conformation, which is consistent with reported *exo* conformational form with the C=O group turns away from benzene ring (Kim *et al.*, 1976). All bond lengths and angles are normal (Allen *et al.*, 1987). Molecules related by an *c*-axis translation are stacked over each other and stabilized by van de waals (Fig. 2). The stacked columns are linked together *via* an intermolecular hydrogen bond, in which the amine H1 act as a donor to H_2_O O1w atom and H_2_O H1A as a donor to H_2_O O1w atom (Fig. 2 and Table 1).

### Experimental

Acetyl chloride (6 ml) was added dropwise to CH_2_Cl_2_ solution (80 ml) containing 2,3,4,5-tetrahydro-1*H*-1,4-benzodiazepine (14.8 g, 0.1 mol) at ice-water bath. After addition, the reaction temperature was raised to room temperature. The resulting mixture was crashed to ice-water bath after stirring for 5 hrs. The organic layer was separated and dried over MgSO_4_. After filtration, the filtrate was evaporated to give an oil which can be crystallized from acetone to give title compound in 96% yield. Single crystals suitable for X-ray analysis (m.p. 358 K) were obtained by slow evaporation of a ethyl acetate/n-hexane/H_2_O solution at 298 K.

### Refinement

The water H atoms and imine H atom were located from Fourier difference maps and refined subject to an O—H restraint of 0.85Å and N—H 0.88 Å. Other H atoms were introduced at calculated positions (C—H = 0.93–0.97 Å) and refined using a riding model. The isotropic displacement parameters of all H atoms were set to 1.2 times *U*_eq_ of the parent atoms.

### Crystal data

**Table:**

C_11_H_14_N_2_O·H_2_O	*F*_000_ = 448
*M**_r_* = 208.26	*D*_x_ = 1.248 Mg m^−^^3^
Tetragonal, *P*4(3)	Melting point: 358 K
*a* = 10.8251 (8) Å	Mo *K*α radiation λ = 0.71073 Å
*b* = 10.8251 (8) Å	Cell parameters from 3047 reflections
*c* = 9.4569 (14) Å	θ = 2.7–26.0º
α = 90º	µ = 0.09 mm^−^^1^
β = 90º	*T* = 296 (2) K
γ = 90º	Block, colourless
*V* = 1108.2 (2) Å^3^	0.20 × 0.20 × 0.15 mm
*Z* = 4	

### Data collection

**Table:**

Bruker SMART CCD diffractometer	972 reflections with *I* > 2σ(*I*)
Radiation source: fine-focus sealed tube	*R*_int_ = 0.016
Monochromator: graphite	θ_max_ = 25.0º
*T* = 296(2) K	θ_min_ = 2.7º
phi and ω scans	*h* = −12→8
Absorption correction: none	*k* = −12→12
5758 measured reflections	*l* = −9→11
1043 independent reflections	

### Refinement

**Table:**

Refinement on *F*^2^	Hydrogen site location: inferred from neighbouring sites
Least-squares matrix: full	H atoms treated by a mixture of independent and constrained refinement
*R*[*F*^2^ > 2σ(*F*^2^)] = 0.035	*w* = 1/[σ^2^(*F*_o_^2^) + (0.0638*P*)^2^ + 0.1073*P*] where *P* = (*F*_o_^2^ + 2*F*_c_^2^)/3
*wR*(*F*^2^) = 0.098	(Δ/σ)_max_ < 0.001
*S* = 1.07	Δρ_max_ = 0.21 e Å^−^^3^
1043 reflections	Δρ_min_ = −0.19 e Å^−^^3^
140 parameters	Extinction correction: none
1 restraint	Absolute structure: Flack (1983)
Primary atom site location: structure-invariant direct methods	Flack parameter: −10 (10)
Secondary atom site location: difference Fourier map	

### Special details

**Table:**

Geometry. All e.s.d.'s (except the e.s.d. in the dihedral angle between two l.s. planes) are estimated using the full covariance matrix. The cell e.s.d.'s are taken into account individually in the estimation of e.s.d.'s in distances, angles and torsion angles; correlations between e.s.d.'s in cell parameters are only used when they are defined by crystal symmetry. An approximate (isotropic) treatment of cell e.s.d.'s is used for estimating e.s.d.'s involving l.s. planes.
Refinement. Refinement of *F*^2^ against ALL reflections. The weighted *R*-factor *wR* and goodness of fit *S* are based on *F*^2^, conventional *R*-factors *R* are based on *F*, with *F* set to zero for negative *F*^2^. The threshold expression of *F*^2^ > σ(*F*^2^) is used only for calculating *R*-factors(gt) *etc*. and is not relevant to the choice of reflections for refinement. *R*-factors based on *F*^2^ are statistically about twice as large as those based on *F*, and *R*- factors based on ALL data will be even larger.

### Fractional atomic coordinates and isotropic or equivalent isotropic displacement parameters (Å^2^)

**Table:**

	*x*	*y*	*z*	*U*_iso_*/*U*_eq_	
O1W	0.40813 (19)	0.43417 (18)	0.4076 (2)	0.0676 (6)	
H1A	0.4427	0.3641	0.3986	0.081*	
H1B	0.3401	0.4358	0.4525	0.081*	
C1	0.6424 (2)	0.9826 (2)	0.3856 (3)	0.0460 (5)	
C2	0.7330 (3)	1.0679 (2)	0.4220 (3)	0.0580 (7)	
H2	0.7462	1.1362	0.3642	0.070*	
C3	0.8037 (3)	1.0533 (3)	0.5422 (3)	0.0701 (8)	
H3	0.8639	1.1112	0.5653	0.084*	
C4	0.7843 (3)	0.9525 (3)	0.6272 (3)	0.0735 (9)	
H4	0.8310	0.9425	0.7090	0.088*	
C5	0.6966 (3)	0.8663 (3)	0.5923 (3)	0.0638 (8)	
H5	0.6853	0.7980	0.6505	0.077*	
C6	0.6240 (2)	0.8789 (2)	0.4716 (3)	0.0490 (6)	
C7	0.5295 (3)	0.7284 (2)	0.3073 (4)	0.0613 (7)	
H7A	0.4792	0.6547	0.3164	0.074*	
H7B	0.6133	0.7023	0.2867	0.074*	
C8	0.4817 (2)	0.8038 (3)	0.1833 (3)	0.0604 (7)	
H8A	0.4760	0.7516	0.1002	0.072*	
H8B	0.3996	0.8343	0.2047	0.072*	
C9	0.5604 (2)	1.0065 (2)	0.2601 (3)	0.0514 (6)	
H9A	0.4761	1.0164	0.2928	0.062*	
H9B	0.5853	1.0835	0.2160	0.062*	
C10	0.6485 (2)	0.8995 (2)	0.0517 (3)	0.0513 (6)	
C11	0.7359 (3)	1.0053 (3)	0.0322 (3)	0.0689 (8)	
H11A	0.7950	0.9850	−0.0398	0.103*	
H11B	0.7783	1.0213	0.1195	0.103*	
H11C	0.6906	1.0776	0.0044	0.103*	
N1	0.5291 (2)	0.7936 (2)	0.4415 (3)	0.0596 (6)	
N2	0.56359 (19)	0.90814 (18)	0.1536 (2)	0.0500 (5)	
O1	0.6565 (2)	0.80907 (18)	−0.0269 (2)	0.0685 (6)	
H1	0.519 (3)	0.747 (4)	0.517 (5)	0.082*	

### Atomic displacement parameters (Å^2^)

**Table:**

	*U*^11^	*U*^22^	*U*^33^	*U*^12^	*U*^13^	*U*^23^
O1W	0.0770 (13)	0.0662 (11)	0.0596 (12)	0.0001 (9)	−0.0051 (10)	0.0138 (10)
C1	0.0511 (12)	0.0457 (12)	0.0412 (12)	0.0081 (10)	0.0042 (10)	−0.0016 (10)
C2	0.0669 (16)	0.0523 (13)	0.0547 (16)	0.0004 (12)	0.0037 (13)	−0.0129 (12)
C3	0.0711 (18)	0.0773 (19)	0.0620 (19)	0.0076 (15)	−0.0121 (15)	−0.0284 (16)
C4	0.0722 (19)	0.101 (2)	0.0476 (16)	0.0341 (18)	−0.0147 (14)	−0.0216 (17)
C5	0.0764 (18)	0.0692 (17)	0.0457 (15)	0.0306 (15)	0.0060 (14)	0.0082 (13)
C6	0.0502 (13)	0.0513 (13)	0.0455 (13)	0.0126 (10)	0.0072 (11)	0.0053 (11)
C7	0.0599 (15)	0.0437 (13)	0.080 (2)	−0.0070 (11)	0.0064 (14)	0.0074 (14)
C8	0.0514 (13)	0.0617 (15)	0.0682 (19)	−0.0105 (11)	−0.0053 (13)	−0.0020 (14)
C9	0.0596 (14)	0.0441 (12)	0.0507 (15)	0.0070 (11)	−0.0044 (12)	0.0048 (11)
C10	0.0640 (15)	0.0508 (13)	0.0392 (12)	−0.0015 (11)	−0.0085 (12)	0.0050 (11)
C11	0.087 (2)	0.0663 (17)	0.0529 (17)	−0.0163 (15)	0.0089 (15)	0.0064 (14)
N1	0.0602 (13)	0.0577 (13)	0.0610 (15)	0.0020 (10)	0.0131 (12)	0.0183 (12)
N2	0.0582 (12)	0.0469 (11)	0.0449 (11)	−0.0001 (9)	−0.0078 (10)	0.0040 (9)
O1	0.0907 (14)	0.0624 (11)	0.0525 (11)	−0.0061 (10)	0.0010 (11)	−0.0097 (10)

### Geometric parameters (Å, °)

**Table:**

O1W—H1A	0.8500	C7—H7A	0.9700
O1W—H1B	0.8499	C7—H7B	0.9700
C1—C2	1.390 (4)	C8—N2	1.463 (3)
C1—C6	1.401 (4)	C8—H8A	0.9700
C1—C9	1.505 (3)	C8—H8B	0.9700
C2—C3	1.379 (4)	C9—N2	1.466 (3)
C2—H2	0.9300	C9—H9A	0.9700
C3—C4	1.372 (5)	C9—H9B	0.9700
C3—H3	0.9300	C10—O1	1.233 (3)
C4—C5	1.372 (5)	C10—N2	1.334 (3)
C4—H4	0.9300	C10—C11	1.497 (4)
C5—C6	1.392 (4)	C11—H11A	0.9600
C5—H5	0.9300	C11—H11B	0.9600
C6—N1	1.411 (4)	C11—H11C	0.9600
C7—N1	1.452 (4)	N1—H1	0.88 (4)
C7—C8	1.519 (4)		
			
H1A—O1W—H1B	116.8	N2—C8—H8A	109.5
C2—C1—C6	119.3 (3)	C7—C8—H8A	109.5
C2—C1—C9	119.9 (2)	N2—C8—H8B	109.5
C6—C1—C9	120.8 (2)	C7—C8—H8B	109.5
C3—C2—C1	121.3 (3)	H8A—C8—H8B	108.0
C3—C2—H2	119.4	N2—C9—C1	113.81 (19)
C1—C2—H2	119.4	N2—C9—H9A	108.8
C4—C3—C2	119.3 (3)	C1—C9—H9A	108.8
C4—C3—H3	120.3	N2—C9—H9B	108.8
C2—C3—H3	120.3	C1—C9—H9B	108.8
C5—C4—C3	120.4 (3)	H9A—C9—H9B	107.7
C5—C4—H4	119.8	O1—C10—N2	122.7 (2)
C3—C4—H4	119.8	O1—C10—C11	119.2 (3)
C4—C5—C6	121.4 (3)	N2—C10—C11	118.1 (2)
C4—C5—H5	119.3	C10—C11—H11A	109.5
C6—C5—H5	119.3	C10—C11—H11B	109.5
C5—C6—C1	118.3 (3)	H11A—C11—H11B	109.5
C5—C6—N1	120.8 (2)	C10—C11—H11C	109.5
C1—C6—N1	120.7 (2)	H11A—C11—H11C	109.5
N1—C7—C8	114.4 (2)	H11B—C11—H11C	109.5
N1—C7—H7A	108.7	C6—N1—C7	119.5 (2)
C8—C7—H7A	108.7	C6—N1—H1	107 (3)
N1—C7—H7B	108.7	C7—N1—H1	116 (3)
C8—C7—H7B	108.7	C10—N2—C8	120.1 (2)
H7A—C7—H7B	107.6	C10—N2—C9	124.3 (2)
N2—C8—C7	110.9 (2)	C8—N2—C9	114.5 (2)
			
C6—C1—C2—C3	−1.0 (4)	C6—C1—C9—N2	−60.6 (3)
C9—C1—C2—C3	175.2 (2)	C5—C6—N1—C7	−123.7 (3)
C1—C2—C3—C4	0.1 (4)	C1—C6—N1—C7	60.3 (3)
C2—C3—C4—C5	0.7 (4)	C8—C7—N1—C6	−79.0 (3)
C3—C4—C5—C6	−0.8 (4)	O1—C10—N2—C8	5.0 (4)
C4—C5—C6—C1	−0.1 (4)	C11—C10—N2—C8	−175.7 (3)
C4—C5—C6—N1	−176.2 (3)	O1—C10—N2—C9	172.1 (2)
C2—C1—C6—C5	0.9 (3)	C11—C10—N2—C9	−8.6 (4)
C9—C1—C6—C5	−175.3 (2)	C7—C8—N2—C10	97.6 (3)
C2—C1—C6—N1	177.0 (2)	C7—C8—N2—C9	−70.8 (3)
C9—C1—C6—N1	0.8 (3)	C1—C9—N2—C10	−84.2 (3)
N1—C7—C8—N2	65.5 (3)	C1—C9—N2—C8	83.6 (3)
C2—C1—C9—N2	123.2 (3)		

### Hydrogen-bond geometry (Å, °)

**Table:**

*D*—H···*A*	*D*—H	H···*A*	*D*···*A*	*D*—H···*A*
N1—H1···O1W^i^	0.88 (5)	2.33 (4)	3.163 (3)	158 (4)

Symmetry codes: (i) *y*, −*x*+1, *z*+1/4.
